# Supplementary material for: The evolutionary history and genomics of European blackcap migration
Source: eLife. 2020 Apr 21;9:e54462. doi: 10.7554/eLife.54462 (PMC7173969; doi:10.7554/eLife.54462)
Supplement: Supplementary file 1. — Libraries designated a and b are from the same library preparation but sequenced on two separate lanes. [file elife-54462-supp1.docx]

**Supplementary File 1**. Summary of sequencing data used for ALLPATHS-LG assembly. Libraries with a and b are from the same library preparation but sequenced on two separate lanes.

| Library | Type | Insert (bp) | Raw (Gb) | Used (%) | Used (Gb) | Sequence coverage | Physical coverage |
| --- | --- | --- | --- | --- | --- | --- | --- |
| 1 | Fragment | 174 ± 20 | 30.3 | 83.6 | 25.3 | 15.3 | 13.3 |
| 2a | Fragment | 178 ± 20 | 24.8 | 84.6 | 21.0 | 12.6 | 11.3 |
| 2b | Fragment | 178 ± 20 | 32.3 | 85.3 | 27.6 | 16.5 | 14.8 |
| Total fragment | | | 87.4 |  | 73.9 | 44.4 | 39.4 |
| 3 | Mate | 1,468 ± 223 | 51.31 | 64.8 | 33.2 | 9.3 | 61.4 |
| 4a | Mate | 4,617 ± 406 | 27.24 | 54.0 | 14.7 | 4.1 | 65.9 |
| 4b | Mate | 4,617 ± 406 | 28.05 | 52.3 | 14.7 | 4.1 | 71.4 |
| 5 | Mate | 9,230 ± 978 | 24.46 | 22.4 | 5.5 | 1.5 | 40.5 |
| 6 | Mate | 9,474 ± 805 | 57.44 | 40.1 | 23.0 | 2.1 | 50.7 |
| Total mate | | | 188.5 |  | 91.1 | 21.1 | 289.9 |
| Final assembly | | | 275.9 |  | 165.0 |  |  |
