# Supplementary material for: The evolutionary history and genomics of European blackcap migration
Source: eLife. 2020 Apr 21;9:e54462. doi: 10.7554/eLife.54462 (PMC7173969; doi:10.7554/eLife.54462)
Supplement: Supplementary file 2. — The second ALLPATHS assembly follows the removal of duplicates and contaminants along with gap filling. [file elife-54462-supp2.docx]

**Supplementary File 2**. Assembly statistics at each stage. The second ALLPATHS assembly follows the removal of duplicates and contaminants along with gap filling.

|  | Length | #_seq | N50 | Ns | Busco^a^ (%) | UCE^b^ (%) | UCE^c^ (%) |
| --- | --- | --- | --- | --- | --- | --- | --- |
| ALLPATHS | 1,032,745,135 | 4,071 | 9,614,374 | 3,2284,017 | 92.1 | 98.0 | 98.8 |
| ALLPATHS | 1,031,299,298 | 2,896 | 16,854,067 | 27,685,636 | 93.6 | 98.2 | 98.9 |
| +Bionano | 1,017,107,033 | 96 | 21,997,114 | 33,034,381 | 92.8 | 95.8 | 96.6 |

^a^ percentage of complete BUSCOs (aves_odb9). Full results:

C:93.2%[S:92.1%,D:1.1%],F:3.9%,M:2.9%,n:4915

C:93.6%[S:92.5%,D:1.1%],F:3.7%,M:2.7%,n:4915

C:92.8%[S:91.7%,D:1.1%],F:3.8%,M:3.4%,n:4915

C = complete, S = complete and single-copy, D = duplicated, F = fragmented, M = missing.

^b^ percentage of UCEs identified using whole-genome alignments for three amniotes (chicken, anole and zebra finch, total 5472)

^c^ percentage of amniote UCEs with greater coverage (of 2560)
