# Supplementary material for: The evolutionary history and genomics of European blackcap migration
Source: eLife. 2020 Apr 21;9:e54462. doi: 10.7554/eLife.54462 (PMC7173969; doi:10.7554/eLife.54462)
Supplement: Supplementary file 3. — Mean position and orientation refer to the location and orientation of scaffolds on the flycatcher genome. The last six scaffolds did not hit any of the flycatcher chromosomes. Comparing the annotation of the blackcap and zebra finch genomes suggests they match the indicated chromosomes. [file elife-54462-supp3.docx]

**Supplementary File 3**. Results from satsuma showing which flycatcher chromosome each scaffold in the blackcap reference genome hit. Mean position and orientation refer to the location and orientation of scaffolds on the flycatcher genome. The last 6 scaffolds did not hit any of the flycatcher chromosomes. Comparing the annotation of the blackcap and zebra finch genomes suggests they match the indicated chromosomes.

| Blackcap scaffold | Flycatcher chr | Mean location | Orientation |
| --- | --- | --- | --- |
| Super-Scaffold_33 | chr_1 | 36485 | 1 |
| Super-Scaffold_8 | chr_1 | 1339683 | -1 |
| Super-Scaffold_37 | chr_1 | 4636214 | -1 |
| Super-Scaffold_76 | chr_1 | 8096257 | 1 |
| Super-Scaffold_4 | chr_1 | 10433711 | -1 |
| Super-Scaffold_80 | chr_1 | 11098626 | -1 |
| Super-Scaffold_32 | chr_10 | 998131 | -1 |
| Super-Scaffold_1819 | chr_10 | 2065253 | 1 |
| Super-Scaffold_13 | chr_11 | 575478 | -1 |
| Super-Scaffold_38 | chr_11 | 1568314 | 1 |
| Super-Scaffold_57 | chr_11 | 2074346 | 1 |
| Super-Scaffold_61 | chr_12 | 122961 | 1 |
| Super-Scaffold_89 | chr_12 | 1181156 | 1 |
| Super-Scaffold_100 | chr_13 | 811989 | -1 |
| Super-Scaffold_79 | chr_13 | 1746253 | 1 |
| Super-Scaffold_72 | chr_14 | 869082 | -1 |
| Super-Scaffold_14 | chr_15 | 747687 | -1 |
| Super-Scaffold_26 | chr_17 | 618869 | -1 |
| Super-Scaffold_60 | chr_18 | 30886 | -1 |
| Super-Scaffold_82 | chr_18 | 689775 | 1 |
| Super-Scaffold_66 | chr_19 | 599067 | 1 |
| Super-Scaffold_56 | chr_1A | 916431 | -1 |
| Super-Scaffold_31 | chr_1A | 2191019 | 1 |
| Super-Scaffold_63 | chr_1A | 2340900 | 1 |
| Super-Scaffold_46 | chr_1A | 4867478 | -1 |
| Super-Scaffold_34 | chr_1A | 5221726 | 1 |
| Super-Scaffold_94 | chr_1A | 5486379 | 1 |
| Super-Scaffold_78 | chr_1A | 5863296 | -1 |
| Super-Scaffold_48 | chr_1A | 6119647 | -1 |
| Super-Scaffold_58 | chr_2 | 76694 | -1 |
| Super-Scaffold_40 | chr_2 | 2567093 | 1 |
| Super-Scaffold_30 | chr_2 | 10337890 | 1 |
| Super-Scaffold_23 | chr_20 | 25286 | 1 |
| Super-Scaffold_9 | chr_20 | 90779 | -1 |
| Super-Scaffold_71 | chr_20 | 522682 | -1 |
| Super-Scaffold_20 | chr_20 | 1226651 | 1 |
| Super-Scaffold_110 | chr_21 | 304262 | 1 |
| Super-Scaffold_44 | chr_21 | 743754 | 1 |
| Super-Scaffold_90 | chr_22 | 85418 | 1 |
| Super-Scaffold_27 | chr_22 | 356802 | -1 |
| Super-Scaffold_64 | chr_22 | 379551 | 1 |
| Super-Scaffold_107 | chr_23 | 91984 | 1 |
| Super-Scaffold_98 | chr_23 | 363554 | -1 |
| Super-Scaffold_74 | chr_23 | 669009 | -1 |
| Super-Scaffold_54 | chr_24 | 400871 | 1 |
| Super-Scaffold_25 | chr_25 | 203656 | 1 |
| Super-Scaffold_7 | chr_26 | 149251 | 1 |
| Super-Scaffold_6 | chr_26 | 525390 | -1 |
| Super-Scaffold_5 | chr_27 | 126850 | -1 |
| Super-Scaffold_92 | chr_27 | 336482 | -1 |
| Super-Scaffold_19 | chr_27 | 506654 | 1 |
| Super-Scaffold_51 | chr_28 | 101721 | 1 |
| Super-Scaffold_68 | chr_28 | 407272 | 1 |
| Super-Scaffold_99 | chr_3 | 752368 | -1 |
| Super-Scaffold_24 | chr_3 | 1202731 | 1 |
| Super-Scaffold_18 | chr_3 | 3257995 | -1 |
| Super-Scaffold_17 | chr_3 | 7826899 | 1 |
| Super-Scaffold_70 | chr_4 | 229429 | 1 |
| Super-Scaffold_36 | chr_4 | 695200 | -1 |
| Super-Scaffold_88 | chr_4 | 788400 | -1 |
| Super-Scaffold_10 | chr_4 | 3064426 | 1 |
| Super-Scaffold_65 | chr_4 | 4511879 | 1 |
| Super-Scaffold_35 | chr_4 | 5697803 | -1 |
| Super-Scaffold_12 | chr_4A | 485424 | 1 |
| Super-Scaffold_105 | chr_4A | 1544995 | -1 |
| Super-Scaffold_104 | chr_5 | 466620 | 1 |
| Super-Scaffold_52 | chr_5 | 627439 | -1 |
| Super-Scaffold_1 | chr_5 | 1502460 | -1 |
| Super-Scaffold_55 | chr_5 | 2409007 | 1 |
| Super-Scaffold_47 | chr_5 | 4131534 | -1 |
| Super-Scaffold_73 | chr_5 | 4955029 | 1 |
| Super-Scaffold_50 | chr_6 | 68382 | 1 |
| Super-Scaffold_29 | chr_6 | 322622 | -1 |
| Super-Scaffold_67 | chr_6 | 2085795 | 1 |
| Super-Scaffold_101 | chr_6 | 3689565 | 1 |
| Super-Scaffold_39 | chr_7 | 97585 | 1 |
| Super-Scaffold_83 | chr_7 | 342163 | -1 |
| Super-Scaffold_103 | chr_7 | 2165078 | -1 |
| Super-Scaffold_109 | chr_7 | 3884732 | -1 |
| Super-Scaffold_41 | chr_8 | 1389136 | -1 |
| Super-Scaffold_16 | chr_8 | 2256504 | -1 |
| Super-Scaffold_22 | chr_9 | 709437 | -1 |
| Super-Scaffold_3 | chr_9 | 2046570 | 1 |
| Super-Scaffold_11 | chr_Z | 550092 | 1 |
| Super-Scaffold_75 | chr_Z | 1636321 | -1 |
| Super-Scaffold_43 | chr_Z | 3625804 | -1 |
| Super-Scaffold_93 | chr_Z | 5025976 | 1 |
| Super-Scaffold_69 | chr_Z | 5275119 | -1 |
| Super-Scaffold_49 | chr_Z | 5618693 | -1 |
| Super-Scaffold_28^a^ | chr_Z_random |  |  |
| Super-Scaffold_2^a^ | chr_25 |  |  |
| Super-Scaffold_102^a^ | chr_Un |  |  |
| Super-Scaffold_2172^a^ | chr_Un |  |  |
| Super-Scaffold_42^a^ | chr_Z_random |  |  |
| Super-Scaffold_59^a^ | chr_3_random |  |  |

^a^ based on the zebra finch annotation
