# Supplementary material for: The evolutionary history and genomics of European blackcap migration
Source: eLife. 2020 Apr 21;9:e54462. doi: 10.7554/eLife.54462 (PMC7173969; doi:10.7554/eLife.54462)
Supplement: Supplementary file 6. [file elife-54462-supp6.docx]

**Supplementary File 6 - Additional details on genome assembly and annotation**

***Library construction*** Next-generation sequencing libraries were sequenced on a HiSeq 4000 with paired-end, 100 bp reads. DNA for Bionano was suspended in CSB and embedded in agarose-CSB mold. It was labelled following IrysPrep Reagent Kit protocol using two nicking enzymes (BspQI and BssSI). The sample was then loaded onto IrysChips and run on the Irys imaging instrument.

***ALLPATHS-LG assembly and improvement*** Next-generation sequencing libraries were subsampled to 50x coverage and default parameters in ALLPATHS-LG were used in the assembly algorithm, with a ploidy of 2 and choosing the option haploidify=true. This is a relatively new option for diploid genomes that are polymorphic. It removes polymorphisms from reads following error correction, creating a mixed haploid dataset that allows longer contigs and scaffolds to be constructed (because polymorphisms generally have the effect of fragmenting assemblies). Polymorphisms are added back to the consensus sequence near the end of the process. You must provide ALLPATHS-LG with information on insert-size and standard deviation. We obtained these values by generating an assembly using estimated values, mapping a subset of the reads to this reference using bwa 0.7.6 for fragment libraries(79) and stampy 1.0.23(80) for mate pair libraries. We used picardtools 1.97 (http://broadinstitute.github.io/picard) CollectInsertSizeMetrics to estimate parameters for the fragment libraries and stampy automatically outputs these data for mate pairs.

Additional steps were taken to improve the initial ALLPATHS-LG assembly. First, we identified and softmasked repeats using RepeatMasker open-4.0 (-ggcalc, -species aves(81)). 6.60% of the reference was masked (compared to 7.76%, 9.08% and 7.93% for hooded crow, chicken and zebra finch). Similar to previous assembles of avian genomes, most of the repeat elements were retroelements (5.04%). Next, we identified duplicate scaffolds and contained sequences using bbmap v 35.51 (https://jgi.doe.gov/data-and-tools/bbtools/). We did not find any duplicated scaffolds, but 7 sequences were contained in larger scaffolds (total of 15,625 bp) and removed. Finally, we filled gaps in the assembly using GapCloser v 1.12(82). 45,493 gaps were identified in the assembly, totalling 32,284,017 Ns. We finished 12,079 gaps (10,599 filled with sequences, 47 with zero length and 1,433 with negative length), reducing number of Ns to 26,162,538. We removed contigs that did not blastn to bird targets using the NCBI nucleotide database.

***Super-scaffolding with optical maps*** ALLPATHS contigs less than 70kb in size were excluded from the hybrid assembly constructed using Bionano optical maps. On its own the BspQI map had 1,463 scaffolds, with an N50 of 0.83 Mb and total length of 1,013.85 Mb. When combined with the NGS assembly the number of contigs was reduced to 110 with an N50 of 21.85 Mb and total length of 1,038.58 bp. The BssSI map had 1,181 scaffolds, with an N50 of 0.82 and total length of 824.26 Mb. After re-scaffolding with this map we had our final assembly.

***Annotation*** The *de novo* testis transcriptome used in our annotation was obtained by sequencing mRNAs of 1 young and 4 adult male individuals. Library reads were obtained with mid input of 75 bp paired-end sequencing. From 8 to 35 M reads mapped for each individual with an average of 22 M reads. The assembly was obtained for each individual separately, using TRINITY with default k-mer parameters (k=25) and a minimum contig size of 300 bp. The different individual transcriptomes were merged eliminating transcripts with 95% similarity among individuals to obtain a final transcriptome.

Four cycles of the MAKER pipeline were run as follows: The first cycle included gene prediction exclusively with EXONERATE and all transcripts and transcriptome as evidence. For the second round we obtained a HMM model to train the SNAP gene predictor and an additional run of EXONERATE with all transcript evidence. The third and fourth rounds included an HMM model obtained from the immediately previous cycle to use it into SNAP and the “chicken” HMM model included in Augustus with default settings. In every cycle, only models with initial and stop codons, and > 50AA were included and accepted.

Genes were functionally annotated using blastp and Interproscan. We used blastp against a database of predicted proteins from ensemble (same species as above) with a threshold of 70% similarity and 80% query coverage. Hits under this thresholds, but higher than 50% in both cases, were flagged with a warning annotation. Second, we run Interproscan with default parameters adding GO terms and Pathways and annotations from the following databases: CDD Gene3D Hamap PANTHER Pfam PIRSF PRINTS ProDom ProSitePatterns ProSiteProfiles SMART SUPERFAMILY TIGRFAM and IPR.
