# Supplementary material for: The evolutionary history and genomics of European blackcap migration
Source: eLife. 2020 Apr 21;9:e54462. doi: 10.7554/eLife.54462 (PMC7173969; doi:10.7554/eLife.54462)
Supplement: Transparent reporting form [file elife-54462-transrepform.docx]

***eLife’s* transparent reporting form**

We encourage authors to provide detailed information *within their submission* to facilitate the interpretation and replication of experiments. Authors can upload supporting documentation to indicate the use of appropriate reporting guidelines for health-related research (see EQUATOR Network), life science research (see the BioSharing Information Resource), or the ARRIVE guidelines for reporting work involving animal research. Where applicable, authors should refer to any relevant reporting standards documents in this form.

If you have any questions, please consult our Journal Policies and/or contact us: [editorial@elifesciences.org](about:blank).

**Sample-size estimation**

- You should state whether an appropriate sample size was computed when the study was being designed
- You should state the statistical method of sample size computation and any required assumptions
- If no explicit power analysis was used, you should describe how you decided what sample (replicate) size (number) to use

Please outline where this information can be found within the submission (e.g., sections or figure legends), or explain why this information doesn’t apply to your submission:

Line 20 in Abstract

Line 124, 179, 228 in Results and Discussion

Figure legends

Where possible we included 10 individuals per phenotype, a general minimum rule for population genetic analyses

**Replicates**

- You should report how often each experiment was performed
- You should include a definition of biological versus technical replication
- The data obtained should be provided and sufficient information should be provided to indicate the number of independent biological and/or technical replicates
- If you encountered any outliers, you should describe how these were handled
- Criteria for exclusion/inclusion of data should be clearly stated
- High-throughput sequence data should be uploaded before submission, with a private link for reviewers provided (these are available from both GEO and ArrayExpress)

Please outline where this information can be found within the submission (e.g., sections or figure legends), or explain why this information doesn’t apply to your submission:

Table S4 includes data on the number of individuals included per population; details on why some birds were excluded can be found on lines 179 and 228 of the Results and Discussion. We use existing software and best practices for our analysis which is described in full in the Methods along with information on how data were processed and filtered prior to analysis. We will upload sequencing data to the SRA upon acceptance.

**Statistical reporting**

- Statistical analysis methods should be described and justified
- Raw data should be presented in figures whenever informative to do so (typically when N per group is less than 10)
- For each experiment, you should identify the statistical tests used, exact values of N, definitions of center, methods of multiple test correction, and dispersion and precision measures (e.g., mean, median, SD, SEM, confidence intervals; and, for the major substantive results, a measure of effect size (e.g., Pearson's r, Cohen's d)
- Report exact p-values wherever possible alongside the summary statistics and 95% confidence intervals. These should be reported for all key questions and not only when the p-value is less than 0.05.

Please outline where this information can be found within the submission (e.g., sections or figure legends), or explain why this information doesn’t apply to your submission:

Brief descriptions of methods employed can be found throughout the results/discussion portion of the manuscript. Full descriptions can be found in the methods section. Raw data are presented in figures when possible. Wherever possible we have represented raw data in figures (Fig1bc, Fig2 and associated supplementary figures, Fig 3 and supplementary figures, Fig 4, Fig 5, Fig 6b). Results from statistical analyses can be found in the Results. Exact p-values are provided throughout unless values are less than 0.0001. Most p-values are calculated by the programs employed that do not provide information on CI.

(For large datasets, or papers with a very large number of statistical tests, you may upload a single table file with tests, Ns, etc., with reference to sections in the manuscript.)

**Group allocation**

- Indicate how samples were allocated into experimental groups (in the case of clinical studies, please specify allocation to treatment method); if randomization was used, please also state if restricted randomization was applied
- Indicate if masking was used during group allocation, data collection and/or data analysis

Please outline where this information can be found within the submission (e.g., sections or figure legends), or explain why this information doesn’t apply to your submission:

Table S4 outlines how birds where assigned to different phenotypic classes.

**Additional data files (“source data”)**

- We encourage you to upload relevant additional data files, such as numerical data that are represented as a graph in a figure, or as a summary table
- Where provided, these should be in the most useful format, and they can be uploaded as “Source data” files linked to a main figure or table
- Include model definition files including the full list of parameters used
- Include code used for data analysis (e.g., R, MatLab)
- Avoid stating that data files are “available upon request”

Please indicate the figures or tables for which source data files have been provided:

The majority of analyses were conducted using existing infrastructure precluding the need to make code available. Sequencing data will be uploaded to the SRA upon acceptance and the relevant phenotypic data are available in Table S4.
